# 

**Published:** 1893-08

**Authors:** 


Vol. IX. AUGUST, 1893. No. 2.

LIBRARY
[illegible]
S. G. O.

### TEXAS Medical Journal.

Subscription, $2.00 a Year, in Advance.
Single Copies, 25 Cents.

PUBLISHED AT AUSTIN, TEXAS, BY

F. E. DANIEL, M. D., & S. E. HUDSON, M. D.,
Editors and Proprietors.

Entered at the Postoffice at Austin, Texas, as Second-class Mail Matter.

